# Analysis of the barriers and enablers to implementing lifestyle management practices for women with PCOS in Singapore

**DOI:** 10.1186/s13104-016-2107-2

**Published:** 2016-06-16

**Authors:** Henry Ko, Helena Teede, Lisa Moran

**Affiliations:** SingHealth Centre for Health Services Research, Singapore Health Services Pte Ltd, 20 College Road, The Academia, Discovery Tower, Level 7 Translational and Clinical Research Hub, Singapore, 169856 Singapore; SingHealth and Duke-NUS Academic Medicine Research Institute, Duke-NUS Graduate Medical School, Academia, Singapore Health Services Pte Ltd, 20 College Road, Singapore, 169856 Singapore; NHMRC Clinical Trials Centre, University of Sydney, Levels 4-6, Medical Foundation Building, 92-94 Parramatta Rd, Camperdown, NSW 2050 Australia; Monash Centre for Health Research & Implementation, School of Public Health, Monash University, Level 1, 43-51, Kanooka Grove, Clayton, VIC 3168 Australia; The Robinson Research Institute, University of Adelaide, Norwich Centre, Ground Floor, 55 King William Road, North Adelaide, SA 5006 Australia

**Keywords:** Polycystic ovary syndrome, Lifestyle management, Clinical practice guideline, Barriers, Prioritisation

## Abstract

**Background:**

Polycystic ovary syndrome (PCOS) is a condition that affects women of reproductive age and manifests with adverse reproductive, metabolic and psychological consequences. Evidence-based PCOS guidelines recommend lifestyle management first line for infertility. In Singapore women with PCOS can attend the PCOS Clinic at the Kandang Kerbau Women and Children’s Hospital for infertility treatment. However lifestyle integration into infertility management is currently limited and barriers and enablers to progress remain unclear.

**Methods:**

All PCOS clinic staff undertook semi-structured interviews to investigate perceived barriers for staff and consumers for the integration of lifestyle into infertility management. This study utilised various tools including an 8P Ishikawa diagram model to identify and categorise barriers. A modified Hanlon method was then used to prioritise barriers within the Singaporean context considering organisational, cultural and financial constraints. Propriety, economics, acceptability, resources and legality (PEARL) criteria were also incorporated into this decision-making tool.

**Results:**

In the 8P model, there were five factors contributing to the ‘procedure (consultations and referral processes)’ barrier, one ‘policy (government and hospitals)’ factor, five ‘place’ factors, two ‘product (lifestyle management programme)’ barriers, two ‘people (programme capacity)’ factors, four ‘process (integration)’ factors, three ‘promotion’ barriers and three ‘price’ factors. Of the prioritised barriers, two were identified across each of ‘procedures’, ‘place’, ‘product’ and ‘people’ and four related to ‘processes’. There were no barriers identified that for ‘policies’, ‘promotion’ and ‘price’ that can be addressed.

**Conclusions:**

There is a clear need to integrate lifestyle into infertility management in PCOS, in line with current national and international evidence-based guidelines. The highest priority identified improvement opportunity was to develop a collaborative lifestyle management programme across hospital services. Reductions in variation of delivery and strengthening support within the lifestyle programme are other identified priorities. The strength of this study is that this is the first study to utilise a pragmatic quality improvement method for barriers identification and prioritisation in the area of lifestyle management for women with PCOS. This project identified factors that may provide easy improvements, but also identified some local factors that may be very difficult to change. The major limitation of this study is that it is only looking at the Singapore setting, so may have limited applicability to other countries. However, results from quality improvement projects are meant to be context specific.

**Electronic supplementary material:**

The online version of this article (doi:10.1186/s13104-016-2107-2) contains supplementary material, which is available to authorized users.

## Background

Polycystic ovary syndrome (PCOS) is a condition affecting up to 18 % of reproductive-aged women [1]. It manifests with adverse reproductive (hyperandrogenism, menstrual dysfunction, infertility and pregnancy complications) [1], metabolic (insulin resistance, dyslipidaemia, non-alcoholic fatty liver disease, endothelial dysfunction, early atherosclerosis and increased impaired glucose tolerance, type 2 diabetes mellitus and cardiovascular disease) [2], and psychological (worsened quality of life and increased anxiety and depression) [3]. Overweight and obesity are strongly related to PCOS with higher energy intake and rates of weight gain driven by hormonal and other factors [4, 5]. Obesity then increases the prevalence and severity of reproductive, metabolic and psychological morbidity in PCOS. PCOS has therefore been identified by the Australian National Women’s Health Policy as a key obesity-related reproductive problem [6].

The first national and international, evidence-based clinical practice guideline (CPG) for the management of PCOS was developed by the PCOS Australian Alliance and approved by the Australian National Health and Medical Research Council in 2011 [7]. The CPG was auspiced by the Jean Hailes Foundation for Women’s Health and supported by the consumer advocacy group Polycystic Ovary Syndrome Association of Australia. A key clinical priority of the CPG was lifestyle (diet, exercise or behavioural) management to optimise weight management (defined as the prevention and treatment of excess weight) and the reproductive, metabolic and psychological features of PCOS delivered through multidisciplinary integrated care. Lifestyle management was also prioritised for the treatment of infertility in PCOS.

Obesity is common and is increasing in Singapore with 10.8 % of adults obese (BMI ≥ 30 kg/m^2^) (9.5 % of females) and 23 % in the high risk Asian BMI category (BMI ≥ 27.5 kg/m^2^) (21.4 % of females) [8, 9]. While the incidence of PCOS in Singapore is unclear, the prevalence of Rotterdam defined PCOS internationally spans from 6 to 18 % depending on the BMI, ethnicity and diagnostic criteria used [10, 11]. Both obesity and PCOS contribute to reduced fertility as does advanced maternal age and changing work and lifestyle commitments amongst women. Possible lack of knowledge about these influencing factors including PCOS may be impacting on lower birth rates in Singapore.

The Singaporean government has a focus on increasing the birth rate and has made fertility a national issue. Infertility is commonly treated through assisted reproductive technologies (ART) [including in vitro fertilisation (IVF)]. The 2014 clinical practice guideline released by the Singapore Ministry of Health on management of infertility recommended weight reduction as first line treatment (i.e. before ART) as a good practice point [12], however more specific recommendations, methods and evidence were not elaborated upon. The Singapore Health Promotion Board is a government organisation that actively promotes healthy living and promotes lifestyle advice resulting in a very visible social presence regarding weight management in Singapore.

Given the current evidence in this field, the recommendations of international evidence-based guidelines and the Singapore guidelines and government focus on addressing increasing infertility highlighting the importance of weight reduction, there is increasing acknowledgement of the need to improve the integration of lifestyle management into PCOS clinical infertility management. Most ART clinics in Singapore are located in the major hospitals, with some located in private facilities. One of the major fertility and ART clinics in Singapore is at the Kandang Kerbau Women’s and Children’s Hospital’s (KKH) IVF Clinic. The KKH PCOS Clinic is co-located within the KKH IVF Clinic and provides advice to women with PCOS seeking pregnancy. The clinic consists of medical and allied health staff, including reproductive endocrinologists, dietitians and exercise physiologists. The KKH PCOS Clinic has acknowledged the need to improve the delivery of lifestyle management programmes. Clinicians identified some local problems with the programme, including: (1) a lack of a coordinated approach to integration of lifestyle, (2) a possibly low adoption rate, (3) a low sustainability rate once started, (4) uncertainty around optimal organisation, resourcing and patient support and (5) a lack of a clear follow-up and monitoring process and documentation for those women dropping out. These factors limited the confidence of local providers to integrate a lifestyle focus into infertility management.

A proposal was made to test and translate the recommendations from the rigorously developed Australian PCOS lifestyle management CPG to the Singaporean setting. This project aimed to determine what the key barriers were to women with PCOS taking up and adhering to a lifestyle management programme and to determine what programme adaptations could improve sustainability. As far as the authors are aware this is the first time a formal barriers and enablers analysis for integration of lifestyle management into infertility management for women with PCOS has been published.

## Methods

This project used methodology based on a quality improvement framework. The problems and barriers to optimal implementation of a lifestyle management programme were identified as a first step to change and implementation.

**Fig. 1:**
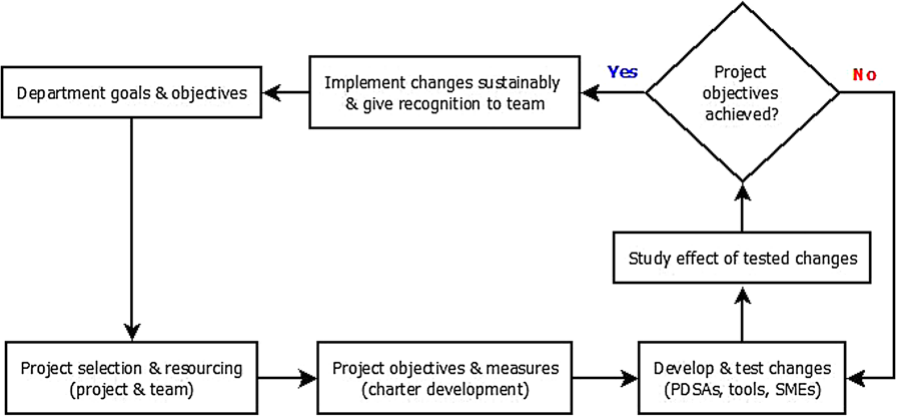
AMI process summary

The pragmatic QI accelerated model for improvement (AMI) methodology promoted by SingHealth department of Clinical Governance and Quality Management, Profound Knowledge Partners Inc [13], and Associates in Process Improvement [14, 15] was used for this exercise (Fig. 1). A key concept in the project selection and resourcing stage are four fundamental ways to implement change:

1. redesign an existing product, process or service,
2. design a new product, process, or service,
3. improve the system as a whole and
4. collaborate and share known solutions to test and modify into other parts of the system.

The activities for project objectives and measures was to document: (1) the project description, (2) the current situation, (3) boundaries, (4) the plan-do-study-act (PDSA) cycles to develop and test changes, (5) identify the goals to be achieved and (6) identify the measures to that can be used to prove success (or failure). PDSA are a QI method where multiple parallel cycles can be used to speed up testing, results, and learning.

The first step was to undertake process mapping or to map the current patient flows and decision-making touch-points for the patient journey within the KKH PCOS and IVF Clinic. Problems and issues were identified through formal and informal face-to-face group and one-on-one semi-structured meetings discussions with all KKH PCOS Clinic clinicians and allied health staff. This included three reproductive medicine and IVF specialists, one endocrinologist, two nutritionists, one nurse, and one exercise physiologist. These staff are representative of all disciplines involved in the KKH PCOS Clinic team with other health care workers. We used the National Institute of Clinical Studies (NICS) guide [16] to identify barriers to evidence uptake by staff during these discussions through techniques including brainstorming, using key informants and interviews and the NICS barrier tool [17]. We also utilised a barrier identification and mitigation (BIM) tool which is used when implementing CPGs as a secondary method for identifying barriers [18]. Due to hospital privacy restrictions, no patients were interviewed for this research.

Problems and issues specific to lifestyle management were structured into an Ishikawa diagram to identify key factors that could be barriers to women taking up and sustaining lifestyle management for their PCOS. An Ishikawa diagram, also known as a fishbone, herringbone, or cause-and-effect diagram, is used to map out the causes of a specific event. It is used in QI research to conceptually and visually group various causes of a problem together into major categories. We used the 8P model commonly used in the service industry as the major categories. The categories are: procedures, policies, place, product, people, processes, price and promotion. ‘Procedures’ related to consultation and referral processes, ‘policies’ related to government and hospital policies, ‘place’ related to Singapore-specific cultural factors, ‘product’ related to the lifestyle management programme, ‘people’ related to the programme capacity and staffing, ‘processes’ related to the integration and coordination of the programme internally and externally to other healthcare services, ‘promotion’ related to how lifestyle management was promoted to stakeholders and ‘price’ related to the financial aspects of PCOS treatments and lifestyle management. The Ishikawa diagram was the main tool to map the barriers and enablers to uptake and sustainability of lifestyle management for women with PCOS in Singapore.

Barriers identified in the Ishikawa diagram were then considered for a priority-setting exercise to determine which barriers should be tackled first with QI methods. We modified the Hanlon method [19] for prioritising health problems to prioritise barriers that we could address pragmatically within the Singaporean setting given the organisational, cultural and financial constraints with only barriers achieving a Hanlon method score >13.5 (the possible median score) considered for future improvement initiatives and QI PDSA development. The modified Hanlon Method rates things based on the sum of three scored criteria—size, seriousness, and effectiveness. This study used a simple ‘yes’, ‘maybe’, or ‘no’ decision to score to what extent each of the identified barriers satisfied each of the modified Hanlon method criteria. The higher the total score (i.e. the more ‘yes’), the more priority the particular barrier has.

We modified the Hanlon Method scoring and problem considerations for our project for the size, seriousness and effectiveness (SSE) criteria (i.e. originally the Hanlon method is for prioritising health problems) to consider the perspective of health service programme delivery and resourcing. The effectiveness criteria ranking for the intervention was ranked by considering the PEARL criteria score, which is further explained later. The Hanlon method for getting the total score for the SSE criteria used the following formula:

$${\text{D }} = \, \left[ {{\text{A }} + \, \left( { 2 { } \times {\text{ B}}} \right) \, \times {\text{ C}}} \right]$$

where: D = priority score A = size of problem ranking, B = seriousness of problem ranking, C = effectiveness of intervention ranking.

Note: according to the Hanlon method, the seriousness of the problem is weighted twice as much as the size of the problem, hence the “2 × B” [19].

For the KKH PCOS Clinic and PCOS in Singapore, the modified Hanlon method scoring considered the following factors:

- ‘Size’: refers to size of the problem for PCOS women.
- ‘Seriousness’: refers to the urgency, demand, economic impact, QoL impact, and adverse health outcomes of this issue for PCOS women.
- ‘Effectiveness’: refers to the predicted effectiveness of any initiatives on addressing the problem. (Note: Effectiveness is based on the PEARL scores).

The maximum possible score is 27. The minimum possible score is zero. The median score is 13.5. It is the modified Hanlon method scores that determine which barriers identified in the Ishikawa diagram are deemed the most important to tackle.

To score the “effectiveness” component of the modified Hanlon method, the authors used a proxy measure to gauge the hypothetical feasibility, importance and impact of tackling the identified barriers. This was done by using the commonly used “PEARL criteria”. The PEARL criteria uses the domains of propriety, economics, acceptability, resources, and legality (PEARL) to identify and rank pragmatic ‘operational’ issues of any barriers and interventions we were proposing to test [19]. The details and explanations on scoring the PEARL criteria are reported in the Additional file 1: Tables S1. Briefly, the analysis rated how each identified barrier could be classified under each PEARL criteria. It is important to note that these criteria were considered primarily from a hospital management and resourcing perspective, and not necessarily from a national public health perspective. For each factor the following questions are considered:

- ‘Propriety’: Is a program for tackling this problem suitable?
- ‘Economics’: Does it make economic sense to address this problem? Are there economic consequences if this problem is not tackled?
- ‘Acceptability’: Will the medical and patient community accept the program? Is it wanted, and by whom?
- ‘Resources’: Is funding available or potentially available for a solution?
- ‘Legality’: Do current laws or corporate management allow proposed solutions to be implemented?

This study used a simple ‘yes’, ‘maybe’, or ‘no’ decision to score to what extent each of the identified barriers satisfied each of the PEARL criteria. Any items that scores at least one “No” will be least prioritised. The more ‘yes’ a barrier had, the higher the total score, and therefore the more important and feasible it may be to tackle that particular barrier with a QI approach. The maximum possible total is 18. The minimum possible total is five. The median score is 11.5.

This pragmatic prioritisation method mostly used information from discussions with the PCOS Clinic staff and understanding of the clinical setting and political/organisational context to inform the rankings, with the scoring providing a semi-quantitative component.

## Results

There are three patients groups referred to the KKH PCOS Clinic from the KKH Infertility or KKH IVF Clinics—patients who have infertility problems (Additional file 1: Figure S1), menstrual problems (Additional file 1: Figure S2), and/or ovulation problems (Additional file 1: Figure S3). Of these, women who are overweight can be recommended to a lifestyle management programme. The lifestyle management programme consists of a set of three diet and one psychology sessions consisting of goal setting and physical activity counselling. There is also follow-up with exercise physiologists, dietitians, and clinicians.

Discussions with key KKH PCOS Clinic staff revealed a generally low uptake of the lifestyle programme as well as low sustainability of the programme. Staff reported that women usually opted for drug treatments and then ART, either immediately or after prematurely dropping out of the lifestyle programme.

**Fig. 2:**
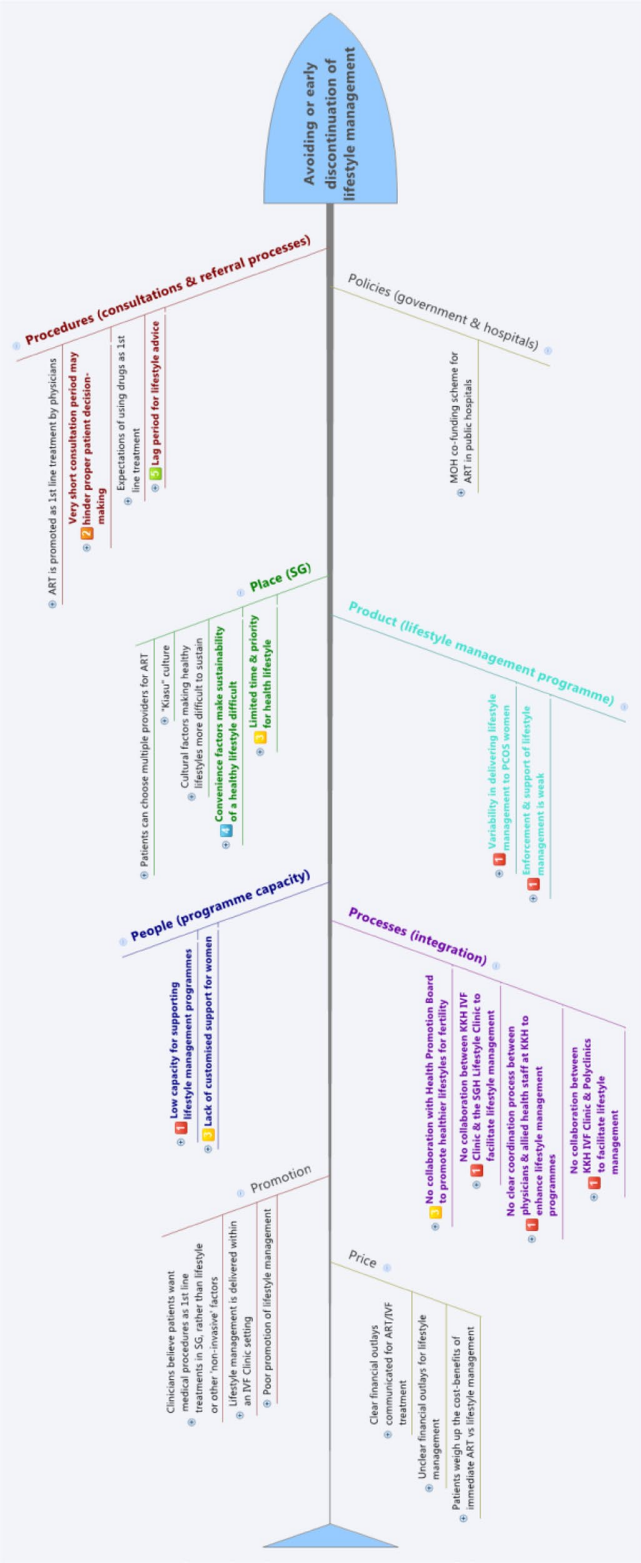
Ishikawa diagram highlighting the ranked barriers to be addressed after the modified Hanlon Method exercise

Using the NICS guide [16] and NICS barrier tool [17] all identified barriers and issues were structured into an Ishikawa diagram “8P model” with the important barriers, determined from the ranking exercise, highlighted (Fig. 2). A more detailed Ishikawa diagram is available in the Supplementary materials online (Additional file 1: Figure S4).

Table 1 shows the key results for the PEARL and modified Hanlon Method ranking exercise, which indicates the which identified barriers may be considered for future QI PDSAs. Of these, two (out of four) barriers have to do with ‘procedures (consultations and referral processes)’, two (out of five) relate to place, two (out of two) barriers relate to ‘product (lifestyle management programme)’, two (out of two) barriers relate to ‘people (programme capacity)’, and four (out of four) barriers relate to ‘processes (integration)’. There were no barriers that were prioritised for ‘policies (government and hospitals)’, ‘promotion’ and ‘price’. Table 1 also identifies the stakeholders that may be affected, the goals of these initial QI PDSAs, and the predicted intensity of the initiatives to be undertaken. More detailed explanations and scoring for each of the identified barriers and how these were ranked for Table 1, can be found in the Additional file 1: Tables S1.

**Table 1:** Key barriers and proposed solutions identified from the PEARL and modified Hanlon Method priority setting exercise

Identified barriers			Proposed solutions		
8P domain	Barrier	“Importance” ranking	Goals of possible next actions and solution/s?	Stakeholder domains (PCOS Clinic operations/health service stakeholders/external stakeholders)	Predicted intensity of work to be undertaken? (high, moderate, low)
Procedures (consultations and referral process)	ART is promoted as 1st line treatment by physicians	Not a top priority	NA	NA	NA
	Very short consultation period may hinder proper patient decision-making	5	Redesign lifestyle management information package to enable clinicians to provide information to patients within a short amount of time. Utilise allied health within the same session?	PCOS clinic operations	High
	Expectations of using drugs as 1st line treatment	Not a top priority	NA	NA	NA
	Lag period for lifestyle advice	2	Redesign operational protocols for patient management and flow. Utilise operations research methods?	PCOS clinic operations	Moderate
Policies (government and hospitals)	Ministry of health (MOH) co-funding scheme for ART in public hospitals	Not a top priority	NA	NA	NA
Place (SG)	Patients can choose multiple providers for ART	Not a top priority	NA	NA	NA
	“Kiasu” culture^a^	Not a top priority	NA	NA	NA
	Cultural factors making healthy lifestyles more difficult to sustain	Not a top priority	NA	NA	NA
	Convenience factors make sustainability of a healthy lifestyle difficult	4	Develop more consistent and coordinated stakeholder collaboration in the lifestyle programme, redesign lifestyle management programme and utilise IT-enabled ‘quantified self’ tools to encourage uptake and sustainability	Health service stakeholders, external stakeholders	High
	Clinicians believe that patients have limited time and priority for healthy lifestyle	3	Develop more consistent and coordinated stakeholder collaboration in the lifestyle programme, redesign lifestyle management programme and utilise IT-enabled ‘quantified self’ tools to encourage uptake and sustainability	Health service stakeholders, external stakeholders	High
Product (lifestyle management programme)	Variability in delivering lifestyle management for PCOS women	1	Develop more consistent and coordinated stakeholder collaboration in the lifestyle programme, redesign lifestyle management programme and utilise IT-enabled ‘quantified self’ tools to encourage uptake and sustainability	PCOS clinic operations, health service stakeholders	High
	Enforcement and support of lifestyle management is weak	1	Develop more consistent and coordinated stakeholder collaboration in the lifestyle programme, redesign lifestyle management programme and utilise IT-enabled ‘quantified self’ tools to encourage uptake and motivation	PCOS clinic operations, health service stakeholders	High
People (programme capacity)	Low capacity for supporting lifestyle management programmes	1	Develop more consistent and coordinated stakeholder collaboration in the lifestyle programme, redesign lifestyle management programme and utilise IT-enabled ‘quantified self’ tools to encourage uptake and motivation	PCOS clinic operations, health service stakeholders	High
	Lack of customised support for women	3	Redesign lifestyle management programme and utilise IT-enabled ‘quantified self’ tools to encourage uptake and motivation	PCOS clinic operations, health service stakeholders	High
Processes (integration)	No collaboration with health promotion board (HPB) to promote healthier lifestyles for improving fertility	3	Explore and design a collaborative programme for lifestyle management for women with PCOS at a national level	Health service stakeholders, external stakeholders	Low
	No collaboration between KKH IVF clinic and the SGH lifestyle clinic to facilitate lifestyle management	1	Explore and design a collaborative programme for lifestyle management for women with PCOS at a health service level	Health service stakeholders	Moderate
	No clear coordination process between physicians and allied health staff at KKH to enhance lifestyle management programmes	1	Redesign operational protocols for patient management and flow. Utilise operations research methods?	PCOS clinic operations, Health service stakeholders	Low
	No collaboration between KKH IVF clinic and polyclinics to facilitate lifestyle management	1	Explore and design a collaborative programme for lifestyle management for women with PCOS at a health service and community level	Health service stakeholders	Moderate
Promotion	Clinicians believe patients want medical procedures as 1st line treatments in SG, rather than lifestyle or other ‘non-invasive’ factors	Not a top priority	NA	NA	NA
	Lifestyle management is delivered within an IVF clinic setting	Not a top priority	NA	NA	NA
	Poor promotion of lifestyle management	Not a top priority	NA	NA	NA
Price	Clear financial outlays communicated for ART/IVF treatment	Not a top priority	NA	NA	NA
	Unclear financial outlays for lifestyle management	Not a top priority	NA	NA	NA
	Patients to evaluate the cost-benefits of immediate ART vs lifestyle management	Not a top priority	NA	NA	NA

“Importance” ranking of “1” indicates the most important and highest priority issue

The intensity of the HPB collaborative work is ‘low’ due to possible HPB leadership role on this proposed national level initiative

^a^ “Kiasu” loosely means “fear of losing” in the Singaporean Chinese community. It is a complex local cultural phenomenon where people have the mindset of not wanting to lose, avoiding risk, and wanting to win

It is noted that the highest priority (i.e. ranked #1) was given to exploring collaborative lifestyle management programmes with the Singapore General Hospital (SGH) Lifestyle Clinic, Polyclinics (i.e. multidisciplinary primary care clinics, which may include allied health and dental care services), and between clinicians and allied health staff in KKH as a matter of improving the current lifestyle management programme “process (integration)”. The other #1 ranked priority was to manage the variability in delivering lifestyle management, and to strengthen and increase the support offered to women in the existing lifestyle programme.

## Discussion

While recent evidence-based guidelines in PCOS highlight the key role of weight management through lifestyle programmes prior to pharmacological management and ART [20–22], there are clear gaps in current care. This study is the first to assess the barriers to implementation of an evidence-based clinical model integrating lifestyle management in infertility treatment in PCOS through identifying prioritising clinician-perceived barriers to integrating lifestyle programmes into infertility management for women with PCOS in Singapore. Barriers included factors relating to ‘procedures (consultations and referral processes)’, ‘place’, ‘product (lifestyle management programme)’, ‘people (programme capacity)’, and ‘processes (integration)’. The barriers of ‘policies (government and hospitals)’, ‘promotion’ and ‘price’ were deemed by study participants as not feasible to tackle without extensive political effort.

Factors that may be relatively easy to tackle are interrelated factors of ‘product’, ‘people’ and ‘process’. The highest priority in this analysis was given to the “process (integration)” barrier of improving the current lifestyle management programme through exploring collaborations with existing programmes and between clinicians and allied health staff as utilised in prior lifestyle management programs and recommended in the CPG [23–25]. From an organisational perspective, synergising and collaborating off existing initiatives would draw upon existing capabilities from each of these stakeholders and eliminate and reduce the duplication of existing programs. The other highly ranked priorities were to reduce the variability in delivering lifestyle management and increase the support offered in the lifestyle management programme. Enhancing multidisciplinary care co-ordination and integration between care providers along the patient journey are key concepts in health service delivery and prioritised by SingHealth and SGH’s Department of Family Medicine and Continuing Care. This could be achieved in this setting through partnership with the SGH LIFE Centre which provides multidisciplinary and coordinated programmes for patients to manage lifestyle-related medical disorders and was Singapore’s first hospital-based integrated and holistic lifestyle improvement facility. Multi-modal initiatives should also be designed to optimise collaborative programmes [26]. For example, existing healthier eating initiatives such as the Healthier Choice Symbol Programme for packaged foods and encouraging the provision of healthier meals at hawker centres and other food outlets is common in Singapore [27].

An identified ‘place’ barrier was the clinician perception that patients have limited time and priority for a healthy lifestyle. Strategies identified for optimising diet and exercise in other populations include the use of IT-enabled lifestyle management tools (i.e. e-health or m-health tools) [28–34]. These may be particularly suitable in this population given the high education levels [35], high smartphone penetration and app usage [36, 37] and existing apps for accessing health information and health monitoring utilised by SingHealth (e.g. Health Buddy, Women and Child HealthPedia, Rheumatoid Arthritis app). These tools may also be preferable compared to face-to-face consultations in this specific population of reproductive-aged women with likely significant personal, work and family priorities [38]. However, testing and auditing of the actual use and effectiveness of these technologies on patients is required before large investments are made.

The barriers that were perceived as relatively difficult to tackle, although not impossible to change, were ‘procedures’ and ‘place’ which were both mainly related to the process of recommending treatment to women. For example, clinical staff thought that there was an expectation amongst Singaporean patients that drugs will be first-line treatment (‘procedure’ factor) and that patients were too busy and preferred to opt for treatment with a medication that may provide immediate results rather than spend months on lifestyle treatments (‘place’ factor). It is likely that the ‘place’ barriers are inherent to the local setting and may include convenience factors that could make sustainability of a healthy lifestyle difficult. Singapore has a high availability of fast food outlets [39] and a culture of eating low-cost street food at hawker centres and reduced home-based food preparation [40]. Opportunities for incidental activity may also be decreased in Singapore due to limited commuting distances related to the dense high-rise built environment and cheap transport. While these barriers cannot be easily addressed unless serious public health promotion efforts are made, a number of efforts are underway to tackle these problems in Singapore [26]. Ethical aspects of implementing lifestyle programme improvements were also highlighted, including a potential disincentive for progressing lifestyle integration into care related to financial incentives for ART clinicians to recommend ART instead of lifestyle management.

QI has been previously used for the assessment of managing obesity-related conditions. However, prior research has to date only described pre-post program implementation results [41, 42], with limited research reporting the specific QI methods used [43]. International databases for use in quality improvement studies include internationally recognised healthcare quality improvement institutions, such as the USA’s Institute for Healthcare Improvement, UK NHS Institute for Innovation and Improvement (which closed in 2013) and Australia’s NSW Agency for Clinical Innovation which contain databases on quality improvement case studies on healthcare initiatives including obesity management [44]. However, the focus has traditionally been for acute care problems rather than on the management of lifestyle and obesity-related conditions with no assessment of PCOS. There is therefore a greater need to utilise and report quality improvement principles used in lifestyle management programs for PCOS because of various complex and interacting factors leading to typically poor attrition and sustainability [45].

The strengths of this study include that it: (1) is the first study to use a QI framework and tools for addressing barriers to implementation of programs in lifestyle management specifically in PCOS and (2) explicitly reports on the methods used to prioritise barriers to address for improvement specific for the Singaporean healthcare setting. One limitation of this study is that the process maps describing the PCOS patient journey may not take into consideration special cases that may occur. The maps were created with input from clinicians about the most common management pathways. The current PEARL and Hanlon prioritisation exercise was performed by “external observers” (i.e. QI/health services research staff) to try to avoid biases and conflicts of interest. Different groups may have differentially prioritised the barriers and enablers. Future research should therefore involve a comparison of these results to those from PCOS Clinic staff, patients, hospital administrators, government officials, or other stakeholders. The seniority and status of ART clinicians compared with allied health staff also needs to be addressed in future research to avoid professional conflicts in the prioritisation of staff opinions. Future research should also engage the key stakeholder groups of patients and health care consumers to identify barriers and assess consistency with clinician-identified barriers, particularly for the elements that patients experience (e.g. ‘place’, ‘promotion’, ‘product’), as well as to help plan, test, and evaluate the strategies for lifestyle management.

The main implications of this study, identified from the QI methodologies, are that: (1) it is important to identify what barriers may or may not be reasonably be address (due to costs, stakeholder buy-in, resource limitations, systems, etc.), (2) financial and resource barriers need to be identified and prioritised from the beginning, (3) government policy and reimbursement “contradictions” may be barriers that are outside the scope of improvement projects. In our setting, government and reimbursement policies were factors that the research team felt were beyond the scope of future QI projects. For this reason, this issue has been ranked as a very low priority area. As the next phase of research, the authors are proposing a number of QI PDSAs to the KKH PCOS Clinic for testing to improve the lifestyle management service offered to patients. This will include addressing the ‘process’ (integration) barriers and to focus on coordinating and building a collaborative PCOS lifestyle management programme that works with other existing lifestyle facilities at SingHealth (e.g. SGH LIFE Centre). Once the collaboration is operational, patients and other stakeholders will be consulted to identify areas to further customise and improve the PCOS lifestyle management programme to optimise the ‘Product’ (i.e. the lifestyle management programme).

## Conclusions

While CPG require implementation and evaluation strategies [46–48] and continuous quality improvement [49, 50] to drive evidence into practice, these activities are often limited and there is no prior research assessing these strategies for women with PCOS in general, and particularly for Singapore. Furthermore, while the recent Singaporean infertility clinical practice guidelines [12] contained recommendations on lifestyle management for PCOS, there was little consideration as to the optimal means of implementation within the current health care system. This research will therefore aid in the practical implementation of these recommendations. This study prioritised key barriers to lifestyle management to address including the exploration of collaborative lifestyle management programmes, reducing variability in delivering lifestyle management and increasing the support offered in the lifestyle management programme.

## Additional file

10.1186/s13104-016-2107-2 KKH PCOS Clinic patient management clinical algorithms.
