# Supplementary material for: Analysis of the barriers and enablers to implementing lifestyle management practices for women with PCOS in Singapore
Source: BMC Res Notes. 2016 Jun 16;9:311. doi: 10.1186/s13104-016-2107-2 (PMC4910192; doi:10.1186/s13104-016-2107-2)
Supplement: Supplementary file 1 — 10.1186/s13104-016-2107-2 KKH PCOS Clinic patient management clinical algorithms. [file 13104_2016_2107_MOESM1_ESM.docx]

**DIAGRAMS**


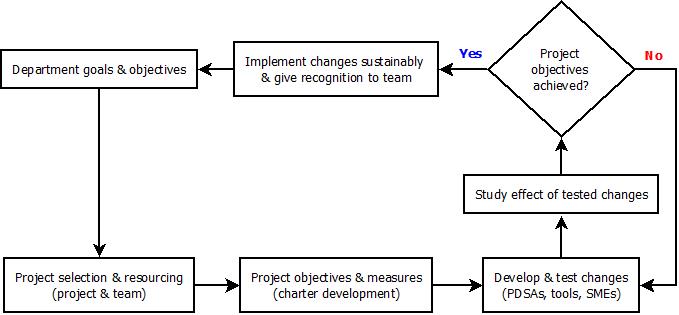


Figure 1. AMI process summary.


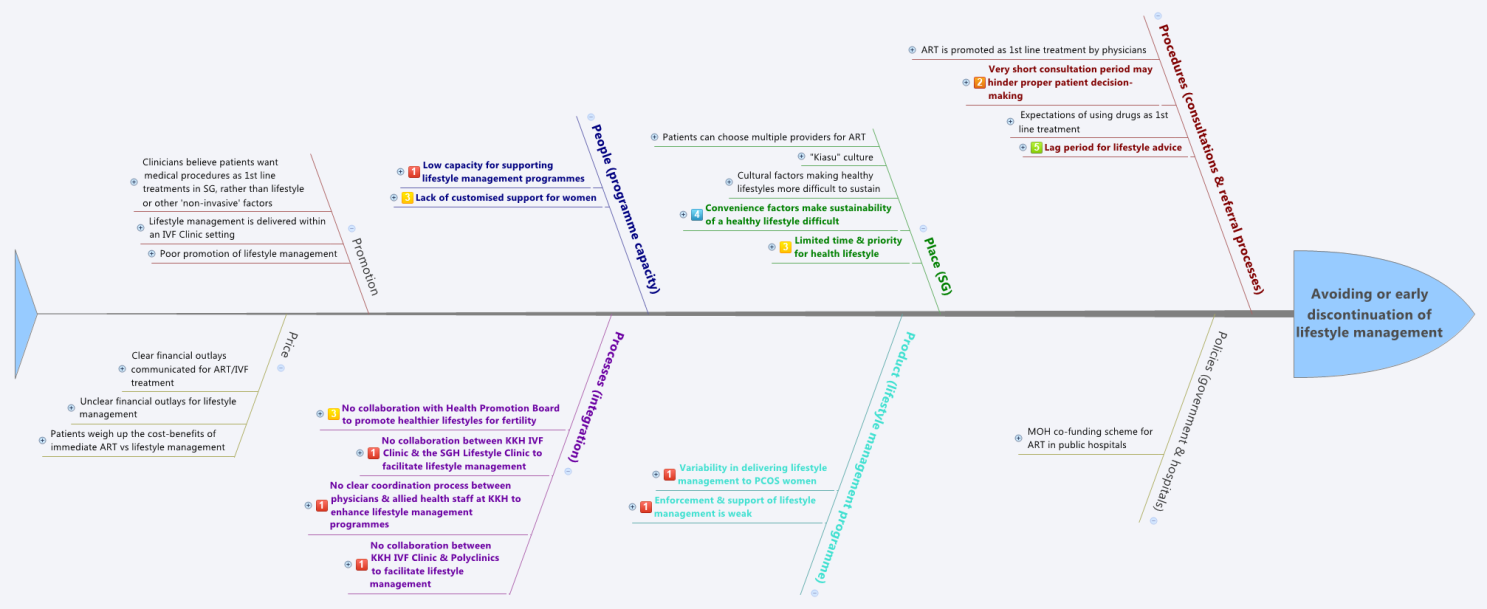


Figure 2. Ishikawa diagram highlighting the ranked barriers to be addressed after the modified Hanlon Method exercise.

**TABLES**

Table 1. Key barriers and proposed solutions identified from the PEARL and modified Hanlon Method priority setting exercise.

| **IDENTIFIED BARRIERS** | | | **PROPOSED SOLUTIONS** | | |
| --- | --- | --- | --- | --- | --- |
| **8P Domain** | **Barrier** | **“Importance” Ranking** | **Goals of possible next actions and solution/s?** | **Stakeholder domains (PCOS Clinic operations / Health service stakeholders / external stakeholders)** | **Predicted intensity of work to be undertaken? (high, moderate, low)** |
| Procedures (consultations and referral process) | ART is promoted as 1st line treatment by physicians | Not a top priority | NA | NA | NA |
|  | Very short consultation period may hinder proper patient decision-making | 5 | Redesign lifestyle management information package to enable clinicians to provide information to patients within a short amount of time. Utilise allied health within the same session? | PCOS Clinic operations | High |
|  | Expectations of using drugs as 1st line treatment | Not a top priority | NA | NA | NA |
|  | Lag period for lifestyle advice | 2 | Redesign operational protocols for patient management and flow. Utilise operations research methods? | PCOS Clinic operations | Moderate |
| Policies (government and hospitals) | Ministry of Health (MOH) co-funding scheme for ART in public hospitals | Not a top priority | NA | NA | NA |
| Place (SG) | Patients can choose multiple providers for ART | Not a top priority | NA | NA | NA |
|  | "Kiasu" culture* | Not a top priority | NA | NA | NA |
|  | Cultural factors making healthy lifestyles more difficult to sustain | Not a top priority | NA | NA | NA |
|  | Convenience factors make sustainability of a healthy lifestyle difficult | 4 | Develop more consistent and coordinated stakeholder collaboration in the lifestyle programme, redesign lifestyle management programme and utilise IT-enabled 'quantified self' tools to encourage uptake and sustainability. | Health service stakeholders, external stakeholders | High |
|  | Clinicians believe that patients have limited time and priority for healthy lifestyle | 3 | Develop more consistent and coordinated stakeholder collaboration in the lifestyle programme, redesign lifestyle management programme and utilise IT-enabled 'quantified self' tools to encourage uptake and sustainability. | Health service stakeholders, external stakeholders | High |
| Product (lifestyle management programme) | Variability in delivering lifestyle management for PCOS women | 1 | Develop more consistent and coordinated stakeholder collaboration in the lifestyle programme, redesign lifestyle management programme and utilise IT-enabled 'quantified self' tools to encourage uptake and sustainability. | PCOS Clinic operations, Health service stakeholders | High |
|  | Enforcement & support of lifestyle management is weak | 1 | Develop more consistent and coordinated stakeholder collaboration in the lifestyle programme, redesign lifestyle management programme and utilise IT-enabled 'quantified self' tools to encourage uptake and motivation. | PCOS Clinic operations, Health service stakeholders | High |
| People (programme capacity) | Low capacity for supporting lifestyle management programmes | 1 | Develop more consistent and coordinated stakeholder collaboration in the lifestyle programme, redesign lifestyle management programme and utilise IT-enabled 'quantified self' tools to encourage uptake and motivation. | PCOS Clinic operations, Health service stakeholders | High |
|  | Lack of customised support for women | 3 | Redesign lifestyle management programme and utilise IT-enabled 'quantified self' tools to encourage uptake and motivation. | PCOS Clinic operations, Health service stakeholders | High |
| Processes (integration) | No collaboration with Health Promotion Board (HPB) to promote healthier lifestyles for improving fertility | 3 | Explore and design a collaborative programme for lifestyle management for women with PCOS at a national level | Health service stakeholders, external stakeholders | Low |
|  | No collaboration between KKH IVF Clinic & the SGH Lifestyle Clinic to facilitate lifestyle management | 1 | Explore and design a collaborative programme for lifestyle management for women with PCOS at a health service level | Health service stakeholders | Moderate |
|  | No clear coordination process between physicians & allied health staff at KKH to enhance lifestyle management programmes | 1 | Redesign operational protocols for patient management and flow. Utilise operations research methods? | PCOS Clinic operations, Health service stakeholders | Low |
|  | No collaboration between KKH IVF Clinic & Polyclinics to facilitate lifestyle management | 1 | Explore and design a collaborative programme for lifestyle management for women with PCOS at a health service and community level | Health service stakeholders | Moderate |
| Promotion | Clinicians believe patients want medical procedures as 1st line treatments in SG, rather than lifestyle or other 'non-invasive' factors | Not a top priority | NA | NA | NA |
|  | Lifestyle management is delivered within an IVF Clinic setting | Not a top priority | NA | NA | NA |
|  | Poor promotion of lifestyle management | Not a top priority | NA | NA | NA |
| Price | Clear financial outlays communicated for ART/IVF treatment | Not a top priority | NA | NA | NA |
|  | Unclear financial outlays for lifestyle management | Not a top priority | NA | NA | NA |
|  | Patients to evaluate the cost-benefits of immediate ART vs lifestyle management | Not a top priority | NA | NA | NA |

NOTES:

“Importance” ranking of “1” indicates the most important and highest priority issue.

*“Kiasu” loosely means “fear of losing” in the Singaporean Chinese community. It is a complex local cultural phenomenon where people have the mindset of not wanting to lose, avoiding risk, and wanting to win.

The intensity of the HPB collaborative work is 'low' due to possible HPB leadership role on this proposed national level initiative.

**SUPPLEMENTAL DIAGRAMS**

**[Current PCOS patient management diagram]**


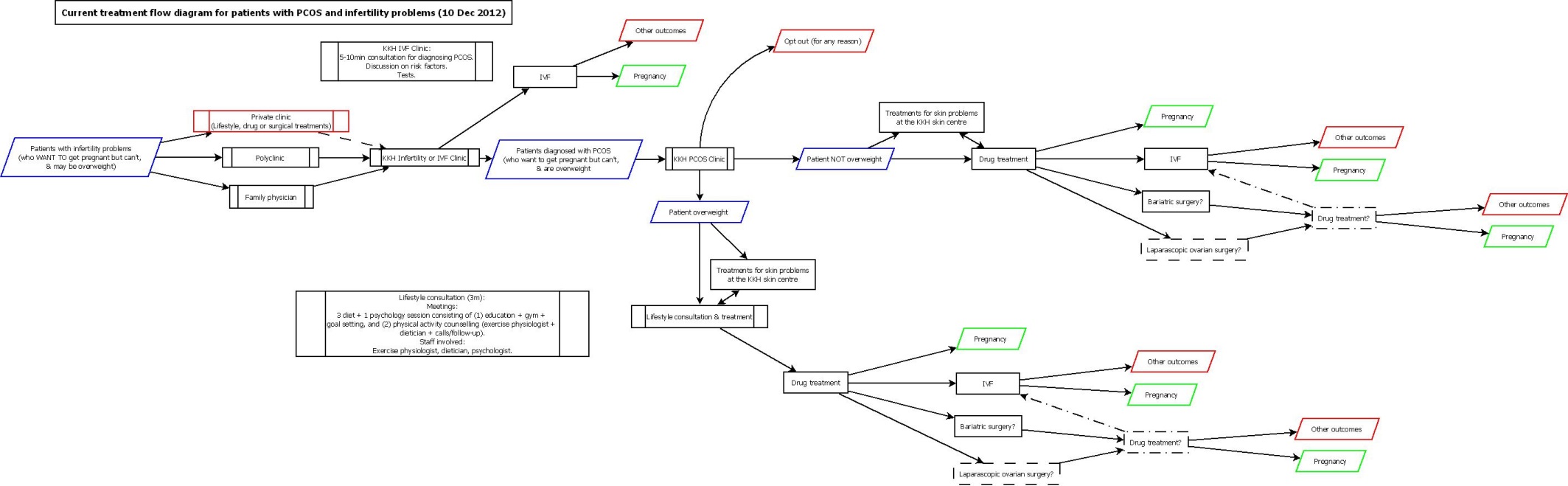


Supplement Figure 1. Current treatment for patients with PCOS and infertility problems at KKH.


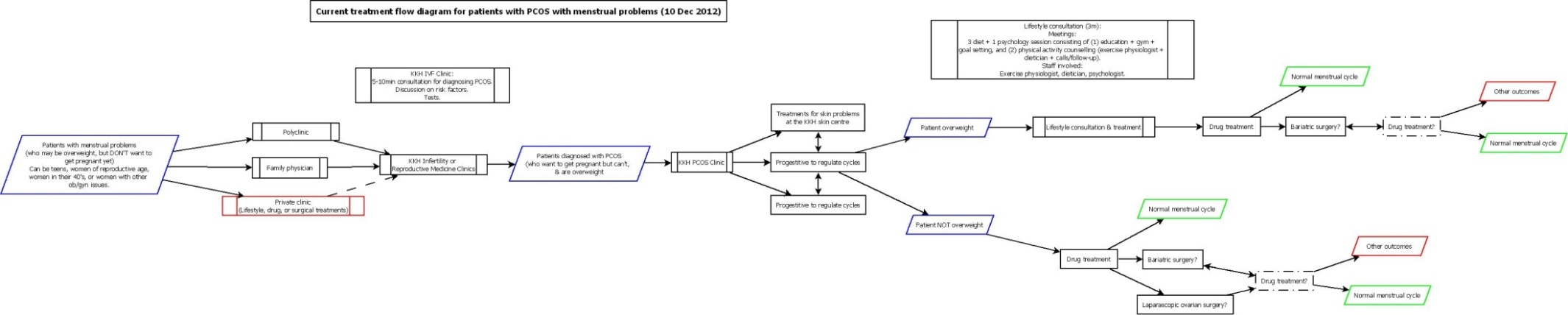


Supplement Figure 2. Current treatment for patients with PCOS and menstrual problems at KKH.


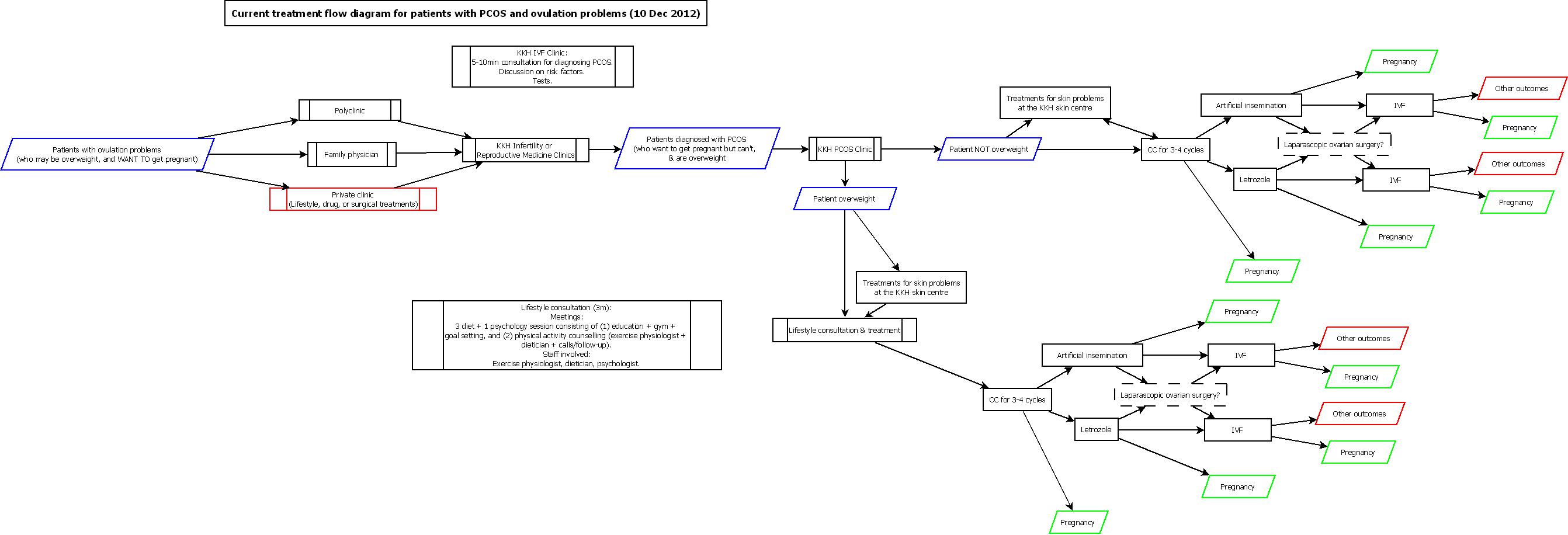


Supplement Figure 3. Current treatment for patients with PCOS and ovulation problems at KKH.

**
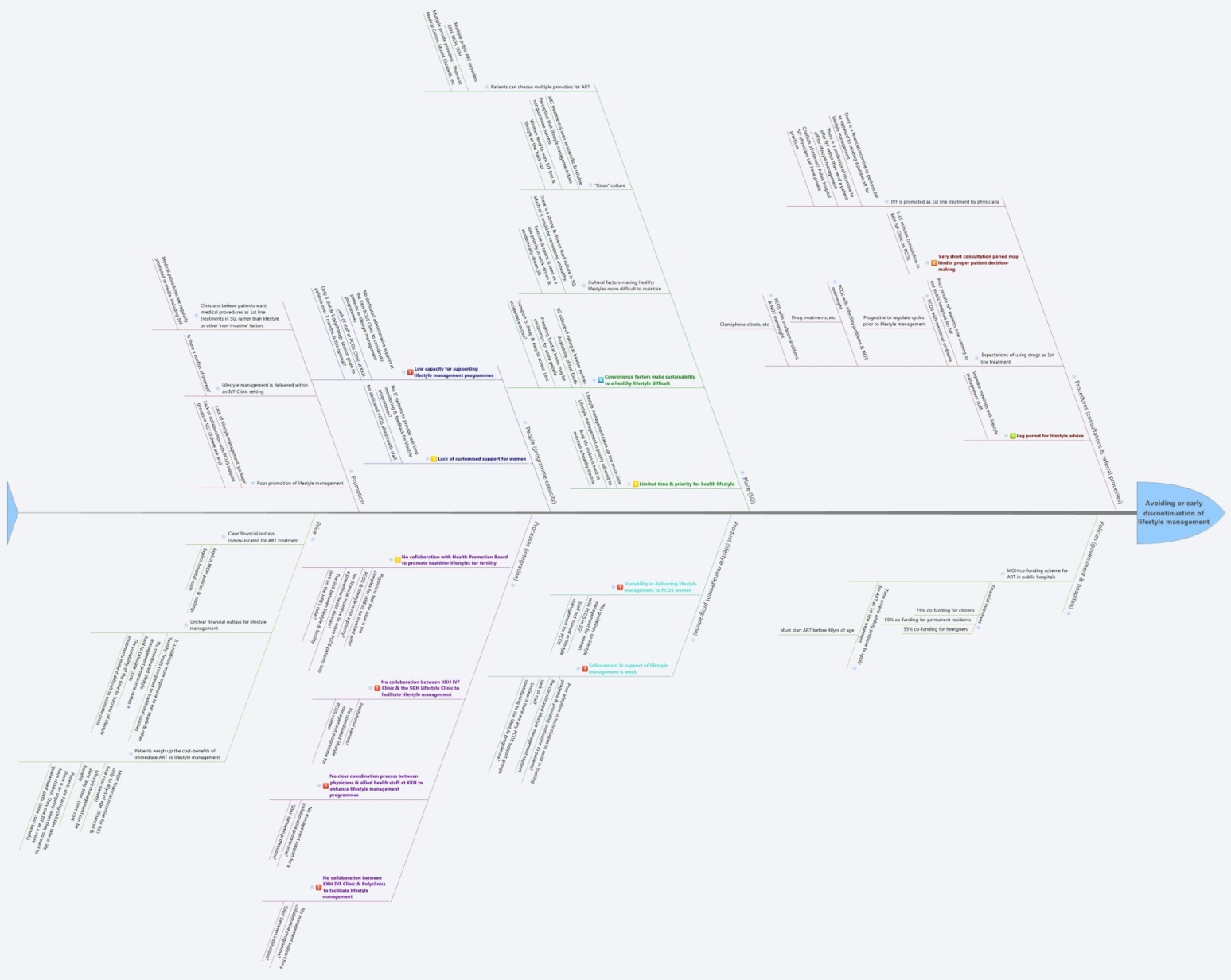
**

Supplement Figure 4. Ishikawa diagram of identified barriers to lifestyle management of patients with PCOS at KKH.

SUPPLEMENTARY TABLES

Table 3. Detailed scoring of the PEARL method for the barriers to tackle

|  |  |  | **PEARL METHOD SCORING (using Hanlon method)** | | | | | |  |
| --- | --- | --- | --- | --- | --- | --- | --- | --- | --- |
| **8P Domain** | **Barrier** | **Contributing reasons** | **Propriety** | **Economics** | **Acceptability** | **Resources** | **Legality** | **TOTALS** | **Comments** |
| Procedures (consultations & referral processes) | IVF is promoted as 1st line treatment by physicians | There is a financial incentive to perform IVF as opposed to sending a patient off for lifestyle management.  There is a professional incentive to offer IVF rather than send a patient off for lifestyle management.  Conflicts of interest? Public hospital IVF physicians can have private practices. | 3 | 1 | 2 | 1 | 2 | 9 | P: Education about the CPG recs may change current practices. The E and R needed to change clinical practices may be high. A and L are against changing current practices as IVF clinicians run the department. |
|  | Very short consultation period may hinder proper patient decision-making | 5-10 minutes consultation in KKH IVF Clinic on PCOS | 3 | 2 | 3 | 2 | 3 | 13 | P: Patients may want longer consultation times. A and L: Longer consultations are feasible to clinicians and clinic management, even if under time pressures. E and R: There is a question mark over how longer consultation times may affect costs and staffing. A solution could be to maximise the efficiency of the consultation by designing a PCOS lifestyle information pack for women (tackles R component). |
|  | Expectations of using drugs as 1st line treatment | Prior private IVF patients now wanting to use public hospitals for IVF.  PCOS with menstrual problems  (Use progestive to regulate cycles prior to lifestyle management)  PCOS with infertility problems & NOT overweight  (Use drug treatments, etc)  PCOS with ovulation problems & NOT overweight  (Use Clomiphene citrate, etc) | 2 | 2 | 2 | 2 | 2 | 10 | This issue links to the expectation by clinicians and patients about using drugs to treat conditions. There may be higher than estimated push-back to change this attitude. |
|  | Lag period for lifestyle management advice | Separate meetings with lifestyle management staff | 3 | 3 | 3 | 2 | 3 | 14 | A solution to streamline or coordinate the rostering and scheduling of lifestyle management sessions may be done using operations engineering research techniques. Given the current poor coordination of various stakeholders, operations research techniques may provide a vast improvement on coordination. |
| Policies (government & hospitals) | MOH co-funding scheme for ART in public hospitals | Financial incentives  (75% co-funding for citizens; 55% co-funding for permanent residents; 35% co-funding for foreigners)  Time criteria adding pressure to apply for ART as 1st line treatment  (Must start ART before 40yrs of age) | 1 | 1 | 1 | 1 | 1 | 5 | Since this is a government policy, there is nothing we can do to tackle this incentive for IVF. |
| Place (SG) | Patients can choose multiple providers for IVF | Multiple public IVF providers - KKH, NUH, SGH  Multiple private providers - Thomson Medical Centre, Mount Elizabeth, etc | 1 | 1 | 1 | 1 | 1 | 5 | Since this is a patient choice issue, possibly even prior to any PCOS diagnosis, we can't really tackle this issue. |
|  | "Kaisu" culture | IVF treatment is seen as scientific & reliable.  Perception that lifestyle management does not guarantee success  Women tend to want IVF first & lifestyle as the 'back-up' | 1 | 1 | 1 | 1 | 3 | 7 | Since this is a cultural phenomenon, which will influence patients' choice to choose things that are deemed efficient and "effective", we can't really tackle this issue. |
|  | Cultural factors making healthy lifestyles more difficult to maintain | There is a strong & diverse food culture in SG. Much of it would be considered unhealthy.  Exercise & sports is seen as a low priority in work-driven & academically-driven SG | 2 | 2 | 2 | 2 | 2 | 10 | The SG HPB is a key player in changing attitudes. SGH Lifestyle clinic are also helping tackle this factor (R factor). However, the E and A are things that unknown and difficult to ascertain. It will be hard to tackle cultural factors on a wide scale (P factor). Tackling this issue is an organisational and government priority, however within the context of the PCOS Clinic, this is not a priority (L factor). |
|  | Convenience factors make adherence to a healthy lifestyle difficult | SG culture of eating at hawker centres  Availability of fast foods  Preparing food at home may be uncommon for some people  Transport is cheap & easy to access. Less incidental exercise? | 2 | 2 | 2 | 3 | 3 | 12 | The SG HPB is a key player in changing attitudes. SGH Lifestyle clinic are also helping tackle this factor (R factor). However, the potential E and A benefits are unknown and difficult to measure compared to behaviours and services that favour convenience. It will be hard to tackle convenience habits on a wide scale (P factor). This issue seems to be a "non-issue" for stakeholders, and therefore may not have much resistance to tackling (L factor). |
|  | Busy lifestyle | Lifestyle management takes up too much time  Lifestyle management is poorly adhered to  Busy life makes it hard to maintain a healthy lifestyle | 2 | 3 | 2 | 3 | 3 | 13 | The SG HPB is a key player in changing attitudes. SGH Lifestyle clinic are also helping tackle this factor, so there are existing resources to tackle this issue (E and R factor). However, the A is something that is unknown and difficult to ascertain. It will be hard to tackle cultural attitudes about being busy on a wide scale (P factor). Tackling this issue is becoming a government priority, however pragmatism and economic factors may work against any favourable policies (L factor). |
| Product (lifestyle management programme) | Variability in delivering lifestyle management to PCOS women | *No guidelines on lifestyle management for women with PCOS in SG*  Staff not trained in lifestyle management for PCOS | 3 | 3 | 3 | 2 | 3 | 14 | The CPG recommendations will guide the more consistent treatment of women with PCOS. Further QI initiatives may drive more consistent lifestyle management delivery. Collaboration with SGH Lifestyle Clinic and HPB may further enhance lifestyle management. |
|  | Enforcement & support of lifestyle management is weak | Poor adoption of technologies to assist in tracking progress & providing motivation to patients?  No coordinated lifestyle management support  Lack of staff  Unclear if there are any PCOS support groups contributing to the lifestyle programme? | 3 | 3 | 3 | 2 | 3 | 14 | QI initiatives may drive more consistent lifestyle management delivery. Utilisation of IT modalities may help to drive a "quantified self" approach to improving compliance and progress tracking. Collaboration with SGH Lifestyle Clinic and HPB may further enhance lifestyle management. |
| People (programme capacity) | Low capacity for supporting lifestyle management programmes | No dedicated administrative support at the KKH PCOS Clinic to coordinate patients or lifestyle management programmes  Lack of staff at PCOS Clinic at KKH  Only 3 diet & 1 psychology session given to patients over 3 months. Is this optimal? | 3 | 2 | 3 | 3 | 3 | 14 | QI initiatives may drive more consistent lifestyle management delivery. Utilisation of IT modalities may help to drive a "quantified self" approach to improving compliance and progress tracking. Collaboration with SGH Lifestyle Clinic and HPB may further enhance lifestyle management. Use of operations research methods may drive more efficient scheduling of staff to support patients. |
|  | Lack of customised support for women | No IT systems to provide real-time monitoring & feedback for lifestyle programmes?  No dedicated PCOS allied health staff | 3 | 2 | 3 | 3 | 3 | 14 | Utilisation of IT modalities may help to drive a "quantified self" approach to improving compliance and progress tracking. Collaboration with SGH Lifestyle Clinic and HPB may further enhance lifestyle management. Developing PCOS-specific lifestyle support materials may help compliance and motivation of women undergoing the lifestyle programme. Collaboration with a Singaporean PCOS patient group (if any?) will definitely help. |
| Processes (integration) | No collaboration with Health Promotion Board to promote healthier lifestyles for fertility | Physicians feel the issue is too complex for HPB to be involved with?  PCOS & lifestyle is not a priority?  No financial incentive to move PCOS patients into a preventive health domain?  The link between lifestyle & fertility isn't on the HPB's radar? | 3 | 3 | 3 | 3 | 2 | 14 | Collaboration may draw on synergies in lifestyle management for better health. Need to arrange organisational collaboration on specific initiatives for this to occur (L factor). |
|  | No collaboration between KKH IVF Clinic & the SGH Lifestyle Clinic to facilitate lifestyle management | Institutional barriers?  No coordinated lifestyle management programme for PCOS women | 3 | 3 | 3 | 3 | 2 | 14 | Collaboration may draw on synergies in lifestyle management for better health. Need to arrange organisational collaboration on specific initiatives for this to occur (L factor). |
|  | No clear coordination process between physicians & allied health staff at KKH to enhance lifestyle management programmes | No management support for a collaborative programme?  'Silos' between professions? | 3 | 3 | 3 | 3 | 2 | 14 | Collaboration may draw on synergies in lifestyle management for better health. Need to arrange organisational collaboration on specific initiatives for this to occur (L factor). May need to draw upon QI and operations research methods to enhance systems of cooperation between groups. |
|  | No collaboration between KKH IVF Clinic & Polyclinics to facilitate lifestyle management | No management support for a collaborative programme?  'Silos' between institutions? | 3 | 3 | 3 | 3 | 2 | 14 | Collaboration may draw on synergies in lifestyle management for better health. Need to arrange organisational collaboration on specific initiatives for this to occur (L factor). |
| Promotion | Patients perceive medical procedures as 1st line treatments in SG, rather than lifestyle or other 'non-invasive' factors | Medical procedures are regularly promoted in media, including IVF | 2 | 2 | 2 | 2 | 2 | 10 | This issue links to the expectation by clinicians and patients about using drugs to treat conditions. There may be higher than estimated push-back to change this attitude. |
|  | Lifestyle management is delivered within an IVF Clinic setting | Is there a conflict of interest? | 1 | 1 | 1 | 1 | 1 | 5 | Nothing can be done about this. |
|  | Poor promotion of lifestyle management | Lack of lifestyle management 'package'  Lack of collaboration with PCOS support groups in SG? (if there are any) | 3 | 3 | 2 | 2 | 2 | 12 | QI initiatives should drive more promotion of lifestyle management. Collaboration with SGH Lifestyle Clinic and HPB may further enhance lifestyle management. More coordination with allied health staff may enhance the uptake of lifestyle management. |
| Price | Clear financial outlays communicated for IVF treatment | Explicit MOH policies & costings  Explicit hospital costs | 1 | 1 | 1 | 1 | 1 | 5 | Nothing can be done about this. |
|  | Unclear financial outlays for lifestyle management | It is relatively more expensive to eat salads & other 'healthy' foods compared to traditional cuisines  No coordinated lifestyle management programme makes it hard to calculate costs  The variability of the time to 'success' of lifestyle treatments make it difficult to estimate costs  The variability of the time to 'success' of lifestyle treatments make it difficult to estimate costs | 2 | 3 | 3 | 2 | 3 | 13 | Telling patients what the costs are might help them make decisions about lifestyle management. However, proper accounting of all the "costs" need to be done, and this may be difficult for lifestyle management. Probably need help from SGH Lifestyle Clinic and HPB to calculate the costs and provide patients with this information. |
|  | Patients weigh up the cost-benefits of immediate ART vs lifestyle management | MOH financial incentive for ART only to 40yrs of age. (financial & time cost-benefit)  Lifestyle management can be done 'any time'. (time cost-benefit)  Patients are having children later in life. There is an urgency when they do want to have children. They see IVF as a more 'guaranteed' path. (time cost-benefit) | 2 | 3 | 2 | 2 | 2 | 11 | It is currently unclear if patients have access to all the information they need to make an informed and considered decision about which treatment path to go on. Because there is no cost estimation of lifestyle treatment currently, we don't think patients are told about all the cost-benefit factors. Having this being told to patients may depend on what the analysis says (possible stakeholder biases?) (P, A, and L factors). |

NOTES:

Scoring: 3 = Yes, 2 = Maybe, 1 = No.

PEARL Test explanation notes:

Propriety: Is a program for tackling this problem suitable?

Economics: Does it make economic sense to address this problem? Are there economic consequences if this problem is not tackled?

Acceptability: Will the medical and patient community accept the program? Is it wanted, and by whom?

Resources: Is funding available or potentially available for a solution?

Legality: Do current laws or corporate management allow proposed solutions to be implemented?

Any items that have at least 1 "No" will be least prioritised. The higher the total score, the more important and feasible it may be to tackle. Maximum possible total = 18. Minimum possible total = 5. Median score = 11.5.

Table 4. Detailed scoring of the modified Hanlon method for the barriers to tackle

|  |  |  | **MODIFIED HANLON METHOD SCORES** | | | |  |
| --- | --- | --- | --- | --- | --- | --- | --- |
| **8P Domain** | **Barrier** | **Contributing reasons** | **Size** | **Seriousness** | **Effectiveness** | **PRIORITY SCORES TOTALS** | **Comments** |
| Procedures (consultations & referral processes) | IVF is promoted as 1st line treatment by physicians | There is a financial incentive to perform IVF as opposed to sending a patient off for lifestyle management.  There is a professional incentive to offer IVF rather than send a patient off for lifestyle management.  Conflicts of interest? Public hospital IVF physicians can have private practices. | 3 | 2 | 1 | 7 | Tackling this issue may require longer term initiatives. There are no clear shorter term initiatives that may change this practice within the SG context. |
|  | Very short consultation period may hinder proper patient decision-making | 5-10 minutes consultation in KKH IVF Clinic on PCOS | 3 | 2 | 2 | 14 | There is no proof that short consultations have negative consequences. |
|  | Expectations of using drugs as 1st line treatment | Prior private IVF patients now wanting to use public hospitals for IVF.  PCOS with menstrual problems  (Use progestive to regulate cycles prior to lifestyle management)  PCOS with infertility problems & NOT overweight  (Use drug treatments, etc)  PCOS with ovulation problems & NOT overweight  (Use Clomiphene citrate, etc) | 2 | 2 | 1 | 6 | Tackling this issue may require longer term initiatives. There are no clear shorter term initiatives that may change this practice within the SG context. |
|  | Lag period for lifestyle management advice | Separate meetings with lifestyle management staff | 3 | 2 | 3 | 21 | It is unclear how lag time affects uptake and compliance. Need to collect data on drop-outs. |
| Policies (government & hospitals) | MOH co-funding scheme for ART in public hospitals | Financial incentives  (75% co-funding for citizens; 55% co-funding for permanent residents; 35% co-funding for foreigners)  Time criteria adding pressure to apply for ART as 1st line treatment  (Must start ART before 40yrs of age) | 3 | 3 | 0 | 0 | No initiative can tackle the MOH policy in the short term. |
| Place (SG) | Patients can choose multiple providers for IVF | Multiple public IVF providers - KKH, NUH, SGH  Multiple private providers - Thomson Medical Centre, Mount Elizabeth, etc | 2 | 2 | 0 | 0 | No initiative can change consumer behaviour to choose in the short term. |
|  | "Kaisu" culture | IVF treatment is seen as scientific & reliable.  Perception that lifestyle management does not guarantee success  Women tend to want IVF first & lifestyle as the 'back-up' | 3 | 2 | 0 | 0 | No initiative can change this cultural phenomenon in the short term. |
|  | Cultural factors making healthy lifestyles more difficult to maintain | There is a strong & diverse food culture in SG. Much of it would be considered unhealthy.  Exercise & sports is seen as a low priority in work-driven & academically-driven SG | 2 | 3 | 1 | 8 | In general, the focus on socio-economic prosperity and competition from a young age perpetuate this attitude and behaviour. |
|  | Convenience factors make adherence to a healthy lifestyle difficult | SG culture of eating at hawker centres  Availability of fast foods  Preparing food at home may be uncommon for some people  Transport is cheap & easy to access. Less incidental exercise? | 2 | 3 | 2 | 16 | SG is built around convenience and catering for people from all cultures and lifestyles. Transport is a very convenience and cheap. SG is small and things are located in close proximity to each other. |
|  | Busy lifestyle | Lifestyle management takes up too much time  Lifestyle management is poorly adhered to  Busy life makes it hard to maintain a healthy lifestyle | 3 | 3 | 2 | 18 | In general, SG lead notoriously busy lifestyles due to the focus on socio-economic prosperity and competition. However, the lower scoring for 'size' is due to the common practice of having foreign maids and helpers in many dwellings. |
| Product (lifestyle management programme) | Variability in delivering lifestyle management to PCOS women | *No guidelines on lifestyle management for women with PCOS in SG*  Staff not trained in lifestyle management for PCOS | 2 | 3 | 3 | 24 | Tackling this issue may be controllable depending on how varied the lifestyle programme is. This is due in part to no PCOS lifestyle programme being in place, poor coordination, and of course no CPG. |
|  | Enforcement & support of lifestyle management is weak | Poor adoption of technologies to assist in tracking progress & providing motivation to patients?  No coordinated lifestyle management support  Lack of staff  Unclear if there are any PCOS support groups contributing to the lifestyle programme? | 2 | 3 | 3 | 24 | This is a key issue to tackle and can be done via testing various initiatives. |
| People (programme capacity) | Low capacity for supporting lifestyle management programmes | No dedicated administrative support at the KKH PCOS Clinic to coordinate patients or lifestyle management programmes  Lack of staff at PCOS Clinic at KKH  Only 3 diet & 1 psychology session given to patients over 3 months. Is this optimal? | 2 | 3 | 3 | 24 | This is a key issue to tackle and can be done via testing various initiatives + better collaboration between stakeholders. |
|  | Lack of customised support for women | No IT systems to provide real-time monitoring & feedback for lifestyle programmes?  No dedicated PCOS allied health staff | 2 | 2 | 3 | 18 | For the 'seriousness' factor it's unclear how customisation may improve outcomes. However, through testing various initiatives, we may be able to determine how important this factor is. |
| Processes (integration) | No collaboration with Health Promotion Board to promote healthier lifestyles for fertility | Physicians feel the issue is too complex for HPB to be involved with?  PCOS & lifestyle is not a priority?  No financial incentive to move PCOS patients into a preventive health domain?  The link between lifestyle & fertility isn't on the HPB's radar? | 2 | 2 | 3 | 18 | This is a key issue to tackle to maintain continuity of care and community support for healthier lifestyles + better collaboration between stakeholders. |
|  | No collaboration between KKH IVF Clinic & the SGH Lifestyle Clinic to facilitate lifestyle management | Institutional barriers?  No coordinated lifestyle management programme for PCOS women | 2 | 3 | 3 | 24 | This is a key issue to tackle to maintain continuity of care and community support for healthier lifestyles + better collaboration between stakeholders. |
|  | No clear coordination process between physicians & allied health staff at KKH to enhance lifestyle management programmes | No management support for a collaborative programme?  'Silos' between professions? | 2 | 3 | 3 | 24 | This is a key issue to tackle to maintain continuity of care and community support for healthier lifestyles + better collaboration between stakeholders. |
|  | No collaboration between KKH IVF Clinic & Polyclinics to facilitate lifestyle management | No management support for a collaborative programme?  'Silos' between institutions? | 2 | 3 | 3 | 24 | This is a key issue to tackle to maintain continuity of care and community support for healthier lifestyles + better collaboration between stakeholders. |
| Promotion | Patients perceive medical procedures as 1st line treatments in SG, rather than lifestyle or other 'non-invasive' factors | Medical procedures are regularly promoted in media, including IVF | 2 | 2 | 1 | 6 | Tackling this issue may require longer term initiatives. There are no clear shorter term initiatives that may change this practice within the SG context. |
|  | Lifestyle management is delivered within an IVF Clinic setting | Is there a conflict of interest? | 3 | 1 | 0 | 0 | No initiative can tackle this issue. |
|  | Poor promotion of lifestyle management | Lack of lifestyle management 'package'  Lack of collaboration with PCOS support groups in SG? (if there are any) | 2 | 2 | 2 | 12 | The seriousness of this issue is unclear as to how it affects lifestyle management of PCOS women. However, it may provide a more favourable environment in which to pursue and comply with lifestyle management regimes. |
| Price | Clear financial outlays communicated for IVF treatment | Explicit MOH policies & costings  Explicit hospital costs | 3 | 3 | 0 | 0 | Nothing to be done for this issue. |
|  | Unclear financial outlays for lifestyle management | It is relatively more expensive to eat salads & other 'healthy' foods compared to traditional cuisines  No coordinated lifestyle management programme makes it hard to calculate costs  The variability of the time to 'success' of lifestyle treatments make it difficult to estimate costs  The variability of the time to 'success' of lifestyle treatments make it difficult to estimate costs | 2 | 2 | 2 | 12 | It's unclear if accurate figures for financial outlays for lifestyle management are communicated to patients (effectiveness factor). It's also unclear how much weighting patients will give to this information in the face of other factors and risks/benefits (seriousness factor). It's also unclear how many patients would consider lifestyle costs an issue (size factor). |
|  | Patients weigh up the cost-benefits of immediate ART vs lifestyle management | MOH financial incentive for ART only to 40yrs of age. (financial & time cost-benefit)  Lifestyle management can be done 'any time'. (time cost-benefit)  Patients are having children later in life. There is an urgency when they do want to have children. They see IVF as a more 'guaranteed' path. (time cost-benefit) | 2 | 2 | 2 | 12 | It's unclear if accurate figures for financial outlays for lifestyle management are communicated to patients (effectiveness factor). It's also unclear how much weighting patients will give to this information in the face of other factors and risks/benefits + MOH incentives (seriousness factor). It's also unclear how many patients would consider lifestyle costs an issue (size factor). |

NOTES:

Scoring: 3 = Yes, 2 = Maybe, 1 = No.

Size: refers to size of the problem for PCOS women

Seriousness: refers to the urgency, demand, economic impact, QoL impact, and adverse health outcomes of this issue for PCOS women

Effectiveness: refers to the predicted effectiveness of any initiatives on addressing the problem. (Note: Effectiveness is based on the PEARL scores)

Maximum possible score = 27. Minimum possible score = 0. Median score = 13.5.
